# Assessment of Genetic Diversity in Seed Plants Based on a Uniform π Criterion

**DOI:** 10.3390/molecules191220113

**Published:** 2014-12-01

**Authors:** Bin Ai, Ming Kang, Hongwen Huang

**Affiliations:** Key Laboratory of Plant Resources Conservation and Sustainable Utilization, South China Botanical Garden, Chinese Academy of Sciences, Guangzhou 510650, China; E-Mails: aibin@scbg.ac.cn (B.A.); mingkang@scbg.ac.cn (M.K.)

**Keywords:** genetic diversity, seed plants, life history traits, breeding system, geographic range, extinction risk

## Abstract

Despite substantial advances in genotyping techniques and massively accumulated data over the past half century, a uniform measurement of neutral genetic diversity derived by different molecular markers across a wide taxonomical range has not yet been formulated. We collected genetic diversity data on seed plants derived by AFLP, allozyme, ISSR, RAPD, SSR and nucleotide sequences, converted expected heterozygosity (*H_e_*) to nucleotide diversity (π), and reassessed the relationship between plant genetic diversity and life history traits or extinction risk. We successfully established a uniform π criterion and developed a comprehensive plant genetic diversity database. The mean population-level and species-level π values across seed plants were 0.00374 (966 taxa, 155 families, 47 orders) and 0.00569 (728 taxa, 130 families, 46 orders), respectively. Significant differences were recovered for breeding system (*p* < 0.001) at the population level and geographic range (*p* = 0.023) at the species level. Selfing taxa had significantly lower π values than outcrossing and mixed-mating taxa, whereas narrowly distributed taxa had significantly lower π values than widely distributed taxa. Despite significant differences between the two extreme threat categories (critically endangered and least concern), the genetic diversity reduction on the way to extinction was difficult to detect in early stages.

## 1. Introduction

According to the neutral theory of molecular evolution, current patterns of neutral genetic diversity that are maintained within species and populations reflect underlying molecular evolutionary processes involving mutation, genetic drift, and gene flow [1,2]. Because of the importance of genetic diversity in evolutionary and conservation genetics [3,4], one fundamental question is how to measure neutral genetic diversity using molecular markers. The capacity of molecular markers to measure genetic diversity has improved greatly over the past half century [5], including allozyme [6], DNA sequencing (since Kreitman) [7], microsatellites (simple sequence repeats, SSR) [8], random amplified polymorphic DNA (RAPD) [9], inter-simple sequence repeats (ISSR) [10] and amplified fragment length polymorphism (AFLP) [11]. Accumulated data and analyses using individual molecular markers have greatly enhanced our understanding of genetic diversity, however, a uniform measurement of neutral genetic diversity that is derived by different molecular markers and capable of being compared across a wide range of different species has not yet been formulated. In recent years, the booming of high-throughput genotyping approach using next-generation sequencing (NGS) [12] has been accompanied by the attenuated application of traditional molecular markers. Thus, it is urgently needed to summarize the previously accumulated genetic diversity data into a meaningful benchmark reference for future use with NGS techniques.

All measured heritable polymorphisms using any molecular marker are derivatives of indirect measurements of nucleotide differences of genomic sequences [5]. Nucleotide diversity (π), which is defined as the average number of nucleotide differences per site between two randomly chosen DNA sequences [13], is a fundamental criterion for measuring genetic diversity [14]. Although DNA sequencing is the direct measurement of π, the data were very limited because of the cost and time constraints in early stages, and instead allozyme, microsatellites, RAPD, ISSR and AFLP have been extensively used for estimating expected heterozygosity (*H_e_*) under Hardy-Weinberg equilibrium [15], the most common parameter used to substitute π thus far. Although several methods have been reported to directly estimate π from AFLP and RAPD data [14,16,17], these methods were impractical for large-scale surveys because of the technical limitation of gel electrophoresis banding complexity. Thus, our first attempt in the present study is to determine whether a large number of reported *H_e_* estimates derived by different molecular markers could be uniformly transformed to π. Few studies have attempted such work, although an evaluation of the correlation between allozyme and DNA sequencing has been performed [4,18].

To date, no solid statistical relationship has been developed between *H*_e_ and π, especially for different markers used in various genetic backgrounds across a wide spectrum of different taxa. The expected nucleotide diversity (π) under neutral equilibrium and infinite sites model is represented by 4*N*_e_μ_0_, where *N*_e_ is the effective population size and μ_0_ is the mutation rate per nucleotide [19]. Alternatively, the expected heterozygosity (*H*_e_) under Hardy-Weinberg equilibrium and the infinite allele model is represented by 4*N*_e_μ/(4*N*_e_μ + 1), where the mutation rate μ refers to the entire protein or amplified fragment [20]. The expected values of both π and *H*_e_ depend on the effective population size and their corresponding mutation rates. Therefore the relationship between π and *H*_e_ is:

(1) $$\text{π = }\frac{{\text{μ}}_{0}H_{\text{e}}}{\text{μ}(1-H_{\text{e}})}$$

and the relationship between *H*_e_ estimates derived by two different markers is

(2) $$\frac{H_{2}}{\left(1-H_{2}\right)}\;=\;\frac{{\text{μ}}_{2}H_{1}}{{\text{μ}}_{1}(1-H_{1})}$$

If we assume that the mutation rate ratios μ_0_/μ and μ_2_/μ_1_ are constant across species and treat *H*_e_/(1 − *H*_e_) as a single parameter, both equations become simple linear formulae, *i.e.*, the correlation and transformation among the estimates derived by different molecular markers could be realized under the assumption of constant mutation rate ratios. The mutation rate ratios could be obtained by performing regression analysis with linear functions through empirical data. In this study, we aimed to test the putative relationship and deduce the *H*_e_-π transformation equations in seed plants, on the basis of a comprehensive compilation of all available data of *H*_e_ estimates derived by different molecular markers.

It is widely accepted that life history traits (taxonomic status, life form, geographic range, breeding system, seed dispersal, successional status, *etc.*) have significant impacts on plant genetic diversity levels based on evidence from several molecular markers, including allozyme [21,22], RAPD [23,24], SSR [24] and DNA sequences [25]. Main conclusions in those reviews and other related studies were quite consistent; however, exceptions have been found for two traits: geographic range at the population level and breeding system at the species level. Geographic range was significantly correlated with population-level *H*_e_ derived by allozyme [21] and SSR [24], but not in the RAPD data [23,24]; the impact of selfing on plant genetic diversity would be reflected at the population level instead of the species level [26,27]. In addition to these inconsistencies, according to the allozyme results, geographic range and breeding system accounted for the largest proportion of genetic variation at the species and population levels, respectively [21]. Thus, we aimed to reassess the association of plant genetic diversity with these two traits at both the species and population levels using a new subset of the compiled database.

There has been a great deal of controversy over the role of genetic factors in extinction risk in the past decades, especially after the “Lande scenario” [28], which has been widely interpreted to mean that most species would be driven to extinction by ecological and demographic factors before genetic factors have time to show impact [29]. Nevertheless, the direct empirical case studies were limited except those assessing *Clarkia pulchella* [30] and *Melitaea cinxia* [31], and most debates primarily relied on the theoretical deductions. Spielman *et al.* used an indirect method by comparing genetic heterozygosity between threatened species and their nonthreatened relatives across animals and plants listed in the International Union for Conservation of Nature (IUCN) Red List of Threatened species [32], but limited plant taxa and no detailed threat categories were included. Thus, reassessment of the association of plant genetic diversity among IUCN categorized plants across a wide taxonomic range was rigorously attempted in the present paper.

The purposes of the present study were three-fold: (1) to establish a uniform π criterion by transforming the *H_e_* estimates using different molecular markers across a wide taxonomic spectrum of seed plants; (2) to reassess the relationship between plant genetic diversity and life history traits or extinction risk; (3) to provide baseline data for future evolutionary studies and conservation practice using NGS techniques.

## 2. Results

**Figure 1:**
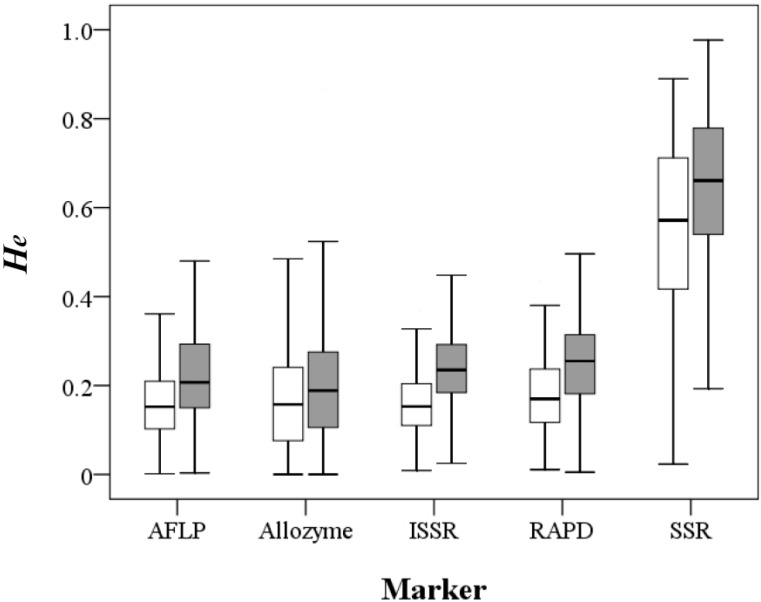
Distribution of the expected heterozygosity values at the population (open box) and species (filled box) levels derived by the five molecular markers.

A total of 1901 records from 1434 papers including 1577 taxa at the species, subspecies or variety level were compiled in the primary literature survey (Table 1). After removing redundancy, the *H_e_* estimates using the five different markers except SSR were quite similar, with mean values ranging 0.159–0.182 at the population level and 0.198–0.246 at the species level, whereas SSR-based *H_e_* estimates were approximately three-fold larger (Mann-Whitney U test, *p* < 0.001), with a mean value of 0.55 at the population level and 0.643 at the species level (Figure 1; Table S1 and Table S2). The π values of 81 records derived by nucleotide sequences ranged from 0.00027 to 0.03318 with a mean value of 0.008 (Table S3).

**Table 1:** The numbers of papers, taxa and records for the five molecular markers and nucleotide sequences included in this study.

Markers	Papers	Taxons	Records
AFLP	244	309	352
Allozyme	383	461	535
ISSR	158	173	183
RAPD	149	171	183
SSR	440	382	535
Sequence	60	81	113
All	1434	1577	1901

**Figure 2:**
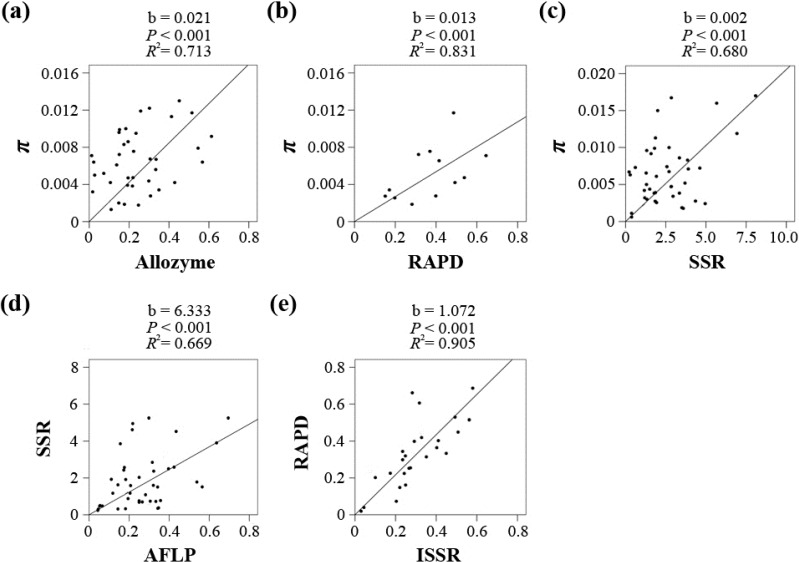
Regression results for each of the five marker pairs using a linear model without intercept, including Sequence/Allozyme (**a**), Sequence/RAPD (**b**), Sequence/SSR (**c**), SSR/AFLP (**d**) and RAPD/ISSR (**e**).

A summary of all pairwise correlation analyses between the π values derived by nucleotide sequences and the *H_e_*/(1 − *H_e_*) values derived by any of the five markers is shown in Table 2, and the taxa chosen for each marker pair are listed in Table S4. A majority of the correlation coefficients were significantly positive (*p* < 0.05), except for three pairs, Sequence/AFLP, Sequence/ISSR and ISSR/SSR, which was most likely due to limited data availability (6, 1 and 7). Five marker pairs (Sequence/Allozyme, Sequence/RAPD, Sequence/SSR, AFLP**/**SSR and RAPD/ISSR) were chosen for the regression analyses to convert *H_e_* to π. The Allozyme-, RAPD-, and SSR-based *H_e_* values were directly converted to π. However, the taxon numbers for the Sequence/AFLP and Sequence/ISSR pairs were too small; therefore, the AFLP- and ISSR-based *H_e_* values were indirectly converted via SSR and RAPD, respectively. As shown in Figure 2 and Table S5, all five coefficients were significant (*p* < 0.001), and the *R^2^* values ranged from 0.669 to 0.905. The linear coefficients for the five markers in the *H_e_*-π conversion equation π = b *H_e_*/(1 − *H_e_*) were 0.021 (Allozyme), 0.013 (RAPD), 0.002 (SSR), 0.012 (AFLP, 6.333 × 0.002) and 0.014 (ISSR, 1.072 × 0.013).

**Table 2:** Summary of the numbers of taxa, Spearman’s coefficients and one-tailed significance tests for correlation analyses among nucleotide sequences and the five molecular markers.

Marker pair	N	rho	*p*
Sequence/AFLP	6	0.600	0.104
Sequence/Allozyme	44	0.288	0.029
Sequence/ISSR	1	/	/
Sequence/RAPD	12	0.538	0.035
Sequence/SSR	49	0.240	0.048
AFLP/Allozyme	24	0.446	0.014
AFLP/ISSR	17	0.418	0.047
AFLP/RAPD	34	0.541	<0.001
AFLP/SSR	47	0.302	0.020
Allozyme/ISSR	9	0.683	0.021
Allozyme/RAPD	39	0.631	<0.001
Allozyme/SSR	67	0.235	0.028
ISSR/RAPD	27	0.825	<0.001
ISSR/SSR	7	0.643	0.060
RAPD/SSR	29	0.335	0.038

All the *H_s_* and *H_t_* values derived by the five markers were converted to π*_s_* and π*_t_* with the deduced regression equations, whereas the π values measured by nucleotide sequences were directly used as π*_t_*. After removing redundancy, 1023 and 807 taxa were obtained in the π*_s_* and π*_t_* datasets, respectively. To eliminate the uncertainty of taxonomic status of cultivated taxa for the follow-up analyses, the cultivated taxa were removed and the taxon numbers were reduced to 966 and 728. The π*_s_* values ranged from 0.0000025 to 0.03285, with a mean value of 0.00374, involving 155 families and 47 orders, whereas the π*_t_* values ranged from 0.0000025 to 0.12900, with a mean value of 0.00569, involving 130 families and 46 orders (Table S6 and Table S7).

**Figure 3:**
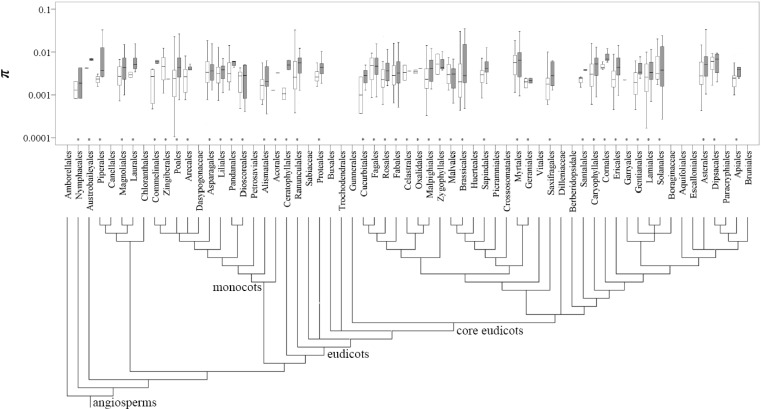
Distribution of the π values at the population (open box) and species (filled box) levels across the angiosperm plants grouped by the orders listed in APG III (2009) (noted with asterisk).

The π*_s_* and π*_t_* values were then mapped onto 43 and 42 orders listed in APG III (2009), respectively (Figure 3). The top three most studied angiosperm families were Asteraceae, Fabaceae and Poaceae, whereas the orders were Asterales, Poales and Lamiales. For the gymnosperm species, the most studied family and order were Pinaceae and Pinales, respectively (Table S7). An approximately 10-fold variation covering two orders of magnitude (0.001 and 0.01) was detected for the mean π*_s_* and π*_t_* values across plant families and orders (Figure 3; Table S7).

The π*_s_* and π*_t_* values from a wide range of taxa were compared among the groups of breeding system, geographic range, and extinction risk (Table 3 and Table S6). One-way ANOVAs revealed significant differences for breeding system (*p* < 0.001) at the population level and geographic range (*p* = 0.023) at the species level, but not for extinction risk at either level. Multiple comparison analyses also revealed significant differences (*p* < 0.05) for several group pairs. Among the breeding system groups, selfing taxa had significantly lower values than other taxa, and asexual taxa had significantly lower values than outcrossing taxa at the population level. However, selfing taxa had significantly lower values than outcrossing and mixed-mating taxa at the species level. Narrowly distributed taxa had significantly lower values than widely distributed taxa at the species level. The CR category had significantly lower values than the LC category at both levels.

**Table 3:** Summary of the population-level (π*_s_*) and species-level (π*_t_*) nucleotide diversity of the sampled taxa grouped by different traits.

Trait	π*_s_*			π*_t_*		
	N	mean	SE	N	mean	SE
Breeding system	* (*p* < 0.001)			NS (*p* = 0.133)		
asexual	102	0.00336 ^b^	0.00310	71	0.00508 ^ab^	0.00390
selfing	70	0.00176 ^c^	0.00196	61	0.00427 ^b^	0.00495
mixed-mating	83	0.00360 ^ab^	0.00363	54	0.00633 ^a^	0.00594
outcrossing	490	0.00418 ^a^	0.00356	365	0.00597 ^a^	0.00641
Geographic range	NS (*p* = 0.368)			* (*p* = 0.023)		
narrow	461	0.00368	0.00324	336	0.00517 ^b^	0.00467
wide	433	0.00389	0.00356	307	0.00652 ^a^	0.00969
Extinction risk	NS (*p* = 0.502)			NS (*p* = 0.493)		
CR	18	0.00241 ^b^	0.00141	17	0.00326 ^b^	0.00231
EN	28	0.00332 ^ab^	0.00283	21	0.00590 ^ab^	0.00701
VU	41	0.00325 ^ab^	0.00314	31	0.00465 ^ab^	0.00318
NT	20	0.00367 ^ab^	0.00263	12	0.00576 ^ab^	0.00421
LC	80	0.00395 ^a^	0.00435	71	0.00685 ^a^	0.01088
All	966	0.00374	0.00343	728	0.00569	0.00730

*Notes*: NS (not significant) and * (*p* < 0.05) stand for the significance of one-way ANOVAs; SE stands for the standard errors; means followed by the same letter in a column are not significantly (*p* < 0.05) different.

## 3. Discussion

The most extensive data compiled and analyzed in this review provided us with valuable insights on the magnitude and variability of genetic diversity in plants (Table S1 and Table S3). Obviously, the results were quite different from what previous authors have concluded. The mean values of Allozyme-based *H_s_* and *H_t_* estimates summarized by Hamrick and Godt [21] were smaller (*H_s_*, 0.113 *vs.* 0.173; *H_t_*, 0.149 *vs.* 0.198). Such differences might be explained by the fact that we collected data accumulated until 2013 much later than those included in Hamrick and Godt [21]. More recently published papers tended to filter the monomorphic markers and use more polymorphic markers. However, larger mean *H_s_* values (AFLP, 0.23 *vs.* 0.162; ISSR, 0.22 *vs.* 0.159; RAPD, 0.22 *vs.* 0.182; SSR, 0.61 *vs.* 0.550) summarized by Nybom *et al.* [24] were likely caused by smaller biased datasets compiled from the four markers (AFLP, 13 *vs.* 247; ISSR, 4 *vs.* 145; RAPD, 60 *vs.* 136; SSR, 104 *vs.* 260). Nevertheless, the comparison among different markers by Nybom *et al.* [24] showed a similar trend that AFLP-, ISSR-, and RAPD-based estimates were quite close whereas SSR-based estimates were three-fold larger.

Our results demonstrated that most of the 15 pairwise correlation coefficients were significantly positive (Table 2), confirming the utility of different molecular markers in population studies. Few assessment of the correlations among different molecular markers had been attempted before this study, except for the relationship between allozyme heterozygosity and nucleotide diversity [4,18]. Pyhäjärvi *et al.* detected a significant (*p* < 0.001) relationship in a dataset from 27 plant species [18], whereas the coefficient was marginally significant (*p* = 0.068) across 22 species studied by Leffler *et al.* [4]. In our study, although no significant correlation was observed for the three marker pairs (Sequence/AFLP, Sequence/ISSR and ISSR/SSR) because of limited data, significant correlations are expected to be recovered when more data become available. Theoretically, genetic variation estimated by molecular markers represents nucleotide differences in genomic sequences, however, the direct numeric relationship might be too difficult to formulate. Several methods were developed for estimating nucleotide diversity from AFLP and RAPD data [14,16,17] on the basis of electrophoresis band profiles, but these methods are impractical for large-scale data compilation. In this study, we confirmed the statistical validity of uniformly transforming the *H_e_* estimates derived by the most commonly used molecular markers with the assumption that the mutation rate ratios of different markers are constant across species. The successful *H_e_*-π conversion by using our deduced regression equations provided us with the first comprehensive plant nucleotide diversity database covering a wide range of plant families and orders.

The association of plant genetic diversity and life history traits (especially geographic range and breeding system) has been of great interest to evolutionary and conservation biologists. One-way ANOVAs in this study showed that genetic diversity revealed as π values was not significantly different among the taxa grouped by geographic range at the population level and by breeding system at the species level (Table 3). Our results suggested that narrowly distributed taxa (mean: 0.00368) were similar to widely distributed taxa (mean: 0.00389) at the population level, which was not in accordance with the conclusion reported for allozyme [21] and SSR [24], but consistence for RAPD [23,24]. One possible explanation was that geographic range is a trait of the whole species other than separate populations. In other words, it might happen that widely distributed taxa have a high level of genetic diversity over the whole species distribution range but a low level in some individual populations with high inter-population differentiation. It has been widely accepted that plant breeding system is a major trait underlying plant genome evolution and molecular diversity, and all of the aforementioned reviews using data from different markers have provided support for this idea. Unexpectedly, the effect of breeding system on genetic diversity was not significant (*p* = 0.133) at the species level in one-way ANOVAs in this study, although selfing taxa (mean: 0.00427) had significantly lower values than outcrossing (mean: 0.00597) and mixed-mating taxa (mean: 0.00633) in the multiple comparison. It was probably due to confounded partition of genetic diversity within and between populations of plant taxa with certain breeding systems. For example, selfer species usually have low intra-population genetic diversity, but high inter-population diversity, such as selfing *Arabidopsis thaliana* compared with its outcrossing relative *A. lyrata* [26,27]. Thus, such confounding attribute may explain that the impact of breeding system on plant genetic diversity was not significant at the species level. Nevertheless, significant differences of genetic diversity were recovered at the population level among the breeding system groups with mean π values ranging from 0.00176 to 0.00418 (Table 3).

The role of genetic factors in extinction risk has been controversial in the past decades [29], especially after Lande demonstrated that ‘demography may usually be of more immediate importance than population genetics in determining the minimum viable size of wild populations’ [28], although Lande has modified his views by readdressing the importance of mutational accumulation in extinction risk [33]. In fact, Lande also mentioned ‘the practical need in conservation for understanding the interaction of demographic and genetic factors in extinction’ [28]. Thus, there was no fundamentally irreconcilable point for the actual controversial debates on the role of genetic factors in increasing extinction risk of small plant populations. To the best of our knowledge, the practical issue is how to detect reductions in genetic diversity while eliminating other confounding factors. Spielman *et al.* compared genetic diversity levels between threatened species and their nonthreatened relatives and found that 77% of threatened species had lower diversity, providing convincing evidence for the importance of genetic factors in conservation [32]. Similar patterns of genetic diversity loss were also reported in mammals [34] and birds [35]. However, the drawbacks in Spielman *et al.* [32] were that a limited number of taxa of 21 angiosperms and 15 gymnosperms were sampled and no detailed threat categories were further investigated.

**Figure 4:**
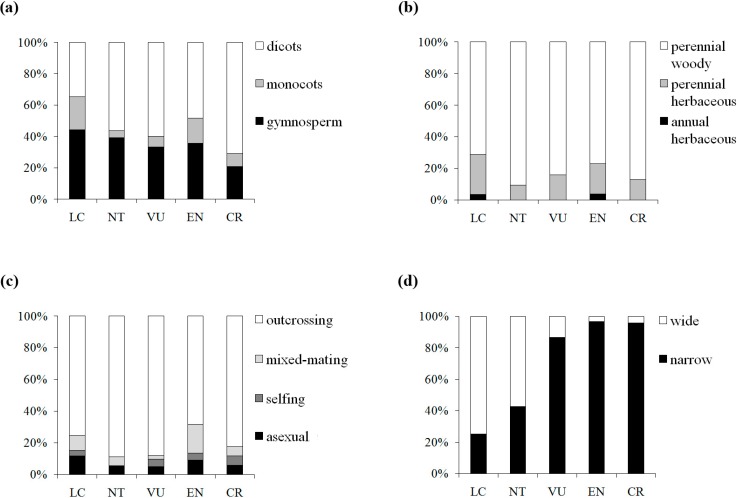
Distribution of the taxon numbers of the five IUCN red list categories, grouped by taxonomic status (**a**), life form (**b**), breeding system (**c**) and geographic range (**d**).

We further pursued this topic by sampling across a wide taxonomic range in seed plants and taking into account five detailed IUCN red list categories. Although one-way ANOVAs showed that genetic diversity levels among the extinction risk groups were not significantly different at either the population or species levels, which might be attributed to the wide range of the π values in the three categories (EN, VU, and NT) with moderate extinction risk, significant differences were detected between CR and LC at the two extremes of the categories in the multiple comparison, suggesting the influence of π on the threat categories ranked by IUCN. CR categorized taxa presented 39% (52%) lower mean π*_s_* (π*_t_*) value than LC categorized taxa, whereas the genetic diversity level was found to decline by 35% in Spielman *et al.* [32]. To examine the relationship, we assigned each category a numerical index from 1 to 5 (1, LC; 2, NT; 3, VU; 4, EN; 5, CR) and detected a marginally significant negative correlation (π*_s_*: Pearson’s *r* = −0.127, *P* = 0.082; π*_t_*: Pearson’s *r* = −0.13, *p* = 0.109) between π and IUCN red list categories, suggesting a weak tendency for genetic diversity to be lower in threat categories with higher extinction risk. Reduction of genetic diversity in plants on the way to extinction might be a gradual and slow process, in which the decline tends to appear far from distinguishable in the preliminary stages. The fuzzy patterns might be caused by confounding factors. By further examining the patterns of four traits (taxonomic status, life form, breeding system and geographic range) associated with the five IUCN red list categories in our dataset, we found a clear shift in elevated extinction risk from wide to narrow geographic range, but no similar pattern was detected for the three other traits (Figure 4). Thus, geographic range would be a good predictor for extinction risk among the plant life history traits. Nevertheless, genetic factors cannot be neglected in the conservation efforts, although the genetic signs of endangerment are difficult to detect in early stages.

It is worth noting that a number of limitations might arise in our data compilation and deduction. First, we assumed that the mutation rate ratios among different markers are constant across plant species. However, this assumption would be violated to some extent because mutation rates vary across species, genomic regions and through evolutionary time. Second, slight differences among several versions of basic equations for calculating *H_e_* estimates [15,36,37,38] might bias the collected data from compiled papers, although this bias was unavoidable for such a large-scale data collection in this study. Finally, proportion of polymorphic loci may result in unexpected confounding deviations for genetic diversity estimates, which has been well documented in the previous reviews [21,23]. Some papers reported estimates of all loci including monomorphic ones, whereas some others only used polymorphic loci by filtering monomorphic ones. In the data collection process, we chose the latter for consistency.

## 4. Experimental Section

### 4.1. Data Collection

Genetic diversity data on seed plants were collected through a literature survey. Papers published before May 2013 that provided expected heterozygosity (*H_e_*) [15,36,37] estimates derived by any of the five commonly used molecular markers (AFLP, Allozyme, ISSR, RAPD and SSR) or nucleotide diversity (π) [13] estimates derived by nuclear gene sequences were included in the data compilation. All available *H_e_* estimates were separately recorded at the population (*H_s_*) or species (*H_t_*) levels, whereas all π values were recorded at the species level. The *H_s_* estimates were averaged from at least two populations for each species, subspecies or variety, whereas the *H_t_* estimates were calculated for all pooled samples. Only the estimates with polymorphic loci were chosen if both estimates of all loci (including monomorphic loci) and polymorphic loci were reported in the same paper, and only the estimates with more represented populations or loci were retained if there was more than one value assessed by the same marker for the same taxon.

### 4.2. Correlation and Regression Analyses, and Data Conversion

Pairwise correlation analyses were performed among the estimates derived by AFLP, Allozyme, ISSR, RAPD, SSR and nucleotide sequences for the same taxa available. The species-level estimates were preferentially included over the population-level estimates. All the *H_e_* values were transformed to *H_e_*/(1 − *H_e_*) prior to the correlation analysis. For numeric conversion from *H_e_* to π, regression analyses between these two values were performed for each taxon. The hypothetic equation was a simple linear function without intercept, π = *b H_e_*/(1 − *H_e_*). If the numbers of the same taxa for the markers were too few and the deduced coefficients were not significant, regression analyses via other markers were used for the *H_e_*-π conversion, *i.e.* π = *b_1_ H_1_*/(1 − *H_1_*) = *b_2_ H_2_*/(1-*H_2_*). Polyploid taxa were excluded for the regression analyses. Subsequently, all the *H_e_* values were converted to π at the population and species levels according to the regression equations. All the π*_s_* and π*_t_* values were gathered and prioritized in the sequential order, sequence > SSR > Allozyme > RAPD > AFLP > ISSR, so that only one value is retained for each taxon. Cultivated taxa (including crops, fruits and vegetables) were then removed for further analysis. Each species was also verified and classified in various taxonomic databases, and angiosperm species followed the APG III version (2009) [39].

### 4.3. Life History Traits and Extinction Risk

For each taxon, data were collected for the important life history traits breeding system (asexual, selfing, mixed-mating, outcrossing) and geographic range (narrow, wide), as well as for the threat category (EX-extinct, EW-extinct in the wild, CR-critically endangered, EN-endangered, VU-vulnerable, NT-near threatened, LC-least concern). For geographic range, “narrow” was equivalent to “endemic” and “narrow” used in Hamrick and Godt [21], whereas “wide” was equivalent to “regional” and “widespread”. The breeding system and geographic range information was obtained from the compiled papers, the USDA-NRCS database (http://plants.usda.gov), pertinent floras or other botanical literature. The threat category information was from the IUCN Red List of Threatened Species version 2013.1 [40]. Mean π values and standard errors were calculated for each group of the three traits at the population and species levels. To test the relationship between genetic diversity and life history traits or extinction risk, one-way analysis of variance (ANOVAs) and multiple comparison analyses were performed to determine the significance levels of the differences in the π values among the groups.

## 5. Conclusions

The present study established a uniform π criterion and developed a comprehensive genetic diversity database across a wide taxonomic spectrum of seed plants and all commonly used genetic markers, thus providing a meaningful benchmark reference for future studies with evolutionary biological and conservation concern. However, questions still remain wide open on the way to solve the old riddle about genetic diversity. The compiled large-scale comprehensive plant genetic diversity database in this study will serve as a solid reference resource for future work. Further discrimination between population-level and species-level with detailed trait hierarchies should be considered in future attempts.

## Supplementary Materials

Supplementary materials can be accessed at: http://www.mdpi.com/1420-3049/19/12/20113/s1.
